# Developing and testing a street audit tool using Google Street View to measure environmental supportiveness for physical activity

**DOI:** 10.1186/1479-5868-10-103

**Published:** 2013-08-23

**Authors:** Pippa Griew, Melvyn Hillsdon, Charlie Foster, Emma Coombes, Andy Jones, Paul Wilkinson

**Affiliations:** 1 Sport and Health Sciences, University of Exeter, Exeter, UK; 2 Department of Public Health, University of Oxford, Oxford, UK; 3 Norwich Medical School, University of East Anglia, Norwich, UK; 4 London School of Hygiene and Tropical Medicine, London, UK

**Keywords:** Physical activity, Walking, Environment, Street audit

## Abstract

**Background:**

Walking for physical activity is associated with substantial health benefits for adults. Increasingly research has focused on associations between walking behaviours and neighbourhood environments including street characteristics such as pavement availability and aesthetics. Nevertheless, objective assessment of street-level data is challenging. This research investigates the reliability of a new street characteristic audit tool designed for use with Google Street View, and assesses levels of agreement between computer-based and on-site auditing.

**Methods:**

The Forty Area STudy street VIEW (FASTVIEW) tool, a Google Street View based audit tool, was developed incorporating nine categories of street characteristics. Using the tool, desk-based audits were conducted by trained researchers across one large UK town during 2011. Both inter and intra-rater reliability were assessed. On-site street audits were also completed to test the criterion validity of the method. All reliability scores were assessed by percentage agreement and the kappa statistic.

**Results:**

Within-rater agreement was high for each category of street characteristic (range: 66.7%-90.0%) and good to high between raters (range: 51.3%-89.1%). A high level of agreement was found between the Google Street View audits and those conducted in-person across the nine categories examined (range: 75.0%-96.7%).

**Conclusion:**

The audit tool was found to provide a reliable and valid measure of street characteristics. The use of Google Street View to capture street characteristic data is recommended as an efficient method that could substantially increase potential for large-scale objective data collection.

## Introduction

Walking is widely promoted as an effective form of physical activity associated with wide ranging health benefits for adults [1]. Current UK guidelines recommend that adults complete at least 150 minutes of moderate intensity activity, such as brisk walking, every week [2]. Nevertheless, the English National Travel Survey 2010 reports that walking trips have declined by 28% since 1995 [3].

Ecological models of health behaviour suggest that environments such as residential neighbourhoods can directly influence physical activity behaviours including walking [4]. However, the objective measurement of environmental variables provides a substantial research challenge. Mapping technologies, such as Geographical Information Systems (GIS), have increased access to objectively characterised data on neighbourhood design and land-use. These data are now regularly utilised in physical activity research and significant associations have been found between walking behaviours and land-use mix, population density and destination proximity [5]. Detailed street-level characteristics such as pavement quality, lighting and aesthetics may additionally be influential. Indeed, walking for both travel and leisure have been significantly associated with individuals’ perceptions of the availability of pavements [6,7] and aesthetics [8]. Objectively measured street-level data, however, are rarely available through mapping databases [9].

A number of street audit tools such as the Systematic Pedestrian And Cycling Environment Scan tool SPACES [10], the Pedestrian Environment Review System (http://www.trl.co.uk) and the Residential Environment Assessment Tool REAT [11] are available to measure the street characteristics hypothesised to influence walking behaviours. Such audit tools are completed in-person by trained researchers and are typically found to provide a valid and reliable measure [10,11]. However, in-person audits are highly time-consuming, have safety issues for personnel and are costly due to the related travel expenditure, thus prohibiting large-scale data collection for the majority of research projects. Recent technological advancements, such as the Google Street View programme (http://www.google.com/maps), might provide an alternative mechanism to traditional in-person street auditing. Google Street View is a freely available web service using video stills of streets and neighbourhoods captured worldwide. The images are displayed to provide continuous panoramic street views that can be navigated along and rotated by 360° allowing the user to virtually walk down any available street from their computer. The use of this desk-based tool, therefore, has the potential to dramatically reduce the resources necessary to complete large-scale assessment of street characteristics.

The reliability of desk-based auditing has previously been investigated by a small number of studies all of which consistently report high levels of agreement between Google Street View and in-person measures [12-14]. Nevertheless, to date, the majority of this research has focused on somewhat homogenous urban areas within the US. Further research is therefore necessary to assess the appropriateness of Google Street View in differing countries and area types where factors such as varying road and pavement width or traffic and building density could potentially impact upon the reliability of the measurement tool.

This study aims to first, test the inter-rater and intra-rater reliability of a newly developed street audit tool designed specifically for use with Google Street View and, second, to test the reliability of this desk-based measure when compared with on-foot street audits across a range of land-use types within the UK. This research forms part of the FAST study (Forty Area STudy). FAST is examining the degree to which built environment characteristics influence adult’s physical activity behaviours across a broad range of social and environmental settings in northwest England. In FAST, we are particularly interested in capturing aspects of the street environment around our participants’ homes that may influence their walking and cycling behaviours, and the street audit tool that we present in this paper, was developed for this purpose.

## Methods

### Street audit tool development

The FASTVIEW audit tool was created in collaboration with the UK Transport Research Laboratory (TRL). The audit tool was based on the Pedestrian Environment Review System (PERS) initially designed by TRL to identify target areas for improving pedestrian access within London and the rest of the UK (http://www.trl.co.uk). PERS and FASTVIEW have different aims and therefore modifications to the original PERS were made to reduce the subjective elements of the tool and to take into account current academic evidence and the expert opinion of the FAST steering committee. For example, an assessment of the housing design and ‘feel’ of a street was replaced with an assessment of housing and street maintenance where auditors were asked to assess factors such as levels of graffiti and litter. The final FASTVIEW tool incorporated nine categories of neighbourhood characteristics (e.g. pavement quality, lighting and safety) with each category including up to three separate factors (e.g. pavement width and spacing of street lights) as displayed in Table 1. Each factor had a number of levels that it was rated on. In Table 1 the levels are listed in the third column and are ordered from more positive through to more negative (researchers wanting access to the tool should contact the corresponding author).

**Table 1:** Walkability categories within the audit tool

**Category**	**Factors**	**Levels**
Pavement width and obstructions	Pavement width (metres)	>3; 2–3; 1–2; <1; no pavement
	Street furniture placement	Aligned to side; poorly placed; N/A*
	Presence of cars parked on the pavement	No cars on pavement; cars on pavement; N/A
Pavement surface quality	Pavement trip hazards	No obvious trip hazards; some trip hazards; N/A
	Pavement surface consistency	Consistent; inconsistent; N/A
	Reinstatements in pavement surface	Not obvious; obvious; N/A
Kerb paving quality	Presence of tactile paving at kerbs	All crossings; >50% of crossings; 50% of crossings; <50% of crossings; no tactile paving; N/A
	Presence of dropped kerbs	All crossings; >50% of crossings; 50% of crossings; <50% of crossings; no dropped kerbs; N/A
Road permeability	Road width (metres)	Pedestrianised street; shared surface street; <6; 6–10; >10
	Obstructions to crossing	No guardrails or parked cars; <50%; 50%; >50%
	Availability of designated crossing points	Quiet residential street; 2+ crossings; 1 crossing; no crossings; N/A
Way finding and legibility	Presence of street name signage	All street names present; >50%; 50%; <50%; none
	Presence of other pedestrian signage	Additional signage; no additional signage
	Presence of landmarks	Landmarks; no landmarks
Lighting	Presence of street lighting	Focussed on pavement; focussed on carriageway; no lighting
	Spacing of street lighting (metres)	20-30; 30–50; >50; N/A
	Likelihood of overnight lighting from nearby buildings	Shop fronts likely to provide light; not likely
Personal security	Evidence of vandalism or graffiti	No evidence; some evidence
	Presence of closed circuit television surveillance	Yes; No
	Informal surveillance from nearby housing	Yes; No
User conflict	Obstruction from bus queues	Yes; No
	Separation between cyclists and pedestrians	Yes; No
	Presence of traffic calming measures	Yes; No
Environment quality	Quality of housing	High quality frontages; low quality frontages
	Presence of trees	Yes; No
	Street maintenance	Clean and well maintained; some litter; some litter and graffiti

* *N/A*, Not Applicable because there was no pavement.

**Figure 1:**
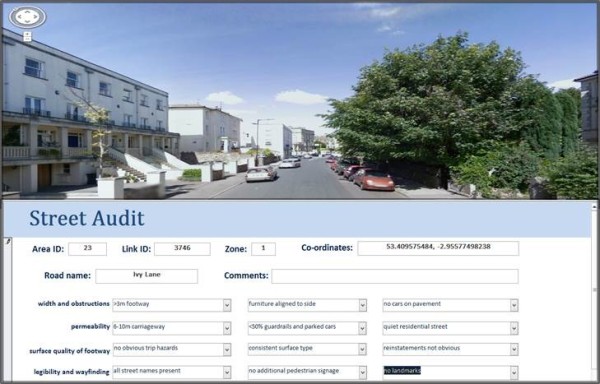
FASTVIEW audit tool.

A data input form was created using Microsoft Access with drop down menu options for all responses. The form was designed to be viewed alongside Google Street View using a computer split screen (Figure 1). Prior to data collection all auditors completed a one-day training course with an experienced auditor from TRL and were provided with a detailed user manual for reference during auditing. For each link both sides of the road were audited. When a road had a pavement on one side only the side with the pavement was audited. For pavement width and street lighting spacing the distance measurement tool in Google Street View was used. A guideline of 10–15 minutes completion time was suggested for each street audit. Both Google Street View and on-street audits took approximately 10 minutes to complete.

### Sampling

**Figure 2:**
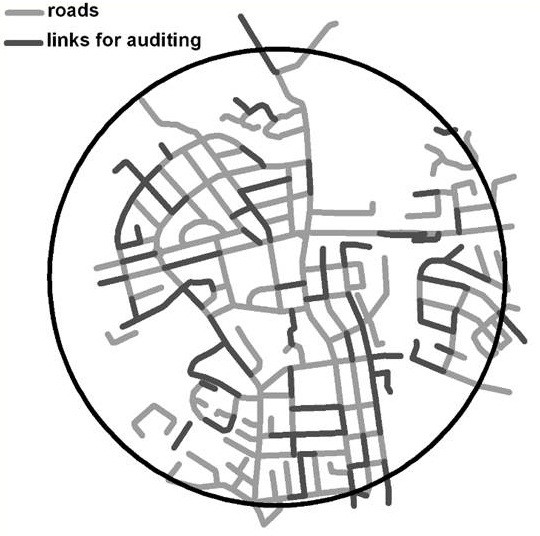
Street network 800 m buffer zone.

Wigan, a large UK town in the North West of England was chosen to test the reliability of the FASTVIEW audit tool due to the large diversity of land-use types (including housing, shopping, parkland and industrial areas) close to the town centre. The population weighted centroid for the town was located using a Geographical Information System (GIS) and the audit area was defined by an 800 m radius (approximately a 10 minute walk) around this centre point (Figure 2). The road network within the study area was mapped in the GIS and divided into road sections termed ‘links’. Where possible these links stretched between road junctions, however in the case of long roads that had few junctions we aimed to maintain homogeneity within links by setting the maximum link length at 300 metres thus minimising the potential for links to straddle different land uses. Consequently long stretches of road were split into a series of links no longer than 300 metres in length. Similarly the minimum link length was set at 50 metres. A total of 216 eligible audit links were identified within the study area, of these 25% (n = 54) were randomly selected for auditing based on recommendations [15]. The 54 links equated to 5.36 kilometres of road network.

### Data reduction and analysis

Each street link was rated as ‘good’ (2), ‘fair’ (1) or ‘poor’ (0) in each of the nine walkability categories based on combined results from the associated factors. For example a street with wide pavements (>3 m), street furniture (e.g. benches and lighting) aligned to the side of the pavement, and with no cars parking on the pavement would get the highest score (2) in the pavement width and obstructions category (see Table 1). In general, if the levels of each category were consistently positive then the category would be rated as good and if they were consistently negative the category would be rated as poor. If the levels were neither consistently good nor poor then the category was rated as fair.

The kappa statistic was chosen for the reliability analysis as it provides a chance-corrected measure of agreement suitable for use with categorical data. However, a recognised limitation of the kappa statistic is that a low kappa co-efficient can be recorded despite high levels of observed agreement dependent upon the prevalence of agreement [16]. Thus results here report and discuss the percentage of observed agreement in conjunction with the calculated kappa co-efficient. Kappa values were interpreted as follows: 0.01-0.20 (slight agreement); 0.21-0.40 (fair agreement); 0.41-0.60 (moderate agreement); 0.61-0.80 (substantial agreement); 0.81-0.99 (almost perfect agreement) [16]. Three reliability tests were conducted: inter-rater, intra-rater and criterion reliability.

#### Intra-rater reliability

To assess the level of agreement over repeated measures each auditor (n = 3) completed desk-based street audits using Google Street View for 20 randomly selected links on two separate occasions. Intra-rater reliability was assessed using Cohens’ kappa statistic.

#### Inter-rater reliability

To assess the level of agreement between researchers all three auditors completed desk based street audits using Google Street View for all of the area links (n = 54). Inter-rater reliability was assessed using Fleiss’ kappa statistic.

#### Criterion reliability

To assess agreement between desk-based audits using Google Street View and in-person street audits, researchers carried out in-person audits for selectively sampled links (n = 30) encompassing a mix of land-use types. The auditing for the reliability study was part of a wider auditing study, with auditors auditing street links using both street view and on-foot visits. The on-foot audits could take place either before or after street view but were always within 6 months of each other. Auditors could not review their scores for one version of the audit prior to undertaking the other. Given the large number of links, it is extremely unlikely that these scores would be recalled. Criterion reliability was assessed using Cohens’ kappa statistic.

All reliability tests were run using STATA version 12.

## Results

Reliability test results are displayed in Table 2. The FASTVIEW tool was found to provide fair to moderate inter and intra-rater reliability over the majority of street characteristic categories and substantial agreement was reported between desk-based audits using Google Street View and those conducted in-person.

**Table 2:** Reliability scores

**Category**	**Inter-rater reliability**		**Intra-rater reliability**		**Criterion reliability**	
	**% Agreement**	**K**	**% Agreement**	**K**	**% Agreement**	**K**
Pavement width and obstructions	88.5	0.597	83.3	0.44	85.0	0.69
Pavement surface quality	51.3	−0.009	70.0	0.48	83.3	0.73
Kerb paving quality	82.7	0.373	86.7	0.45	75.0	0.55
Road permeability	58.3	0.143	73.3	0.48	86.7	0.73
Way finding and legibility	71.8	0.313	86.7	0.66	76.7	0.50
Lighting	57.7	−0.075	86.7	0.60	78.3	0.61
Personal security	79.5	0.445	86.7	0.42	88.3	0.64
User conflict	89.1	0.451	90.0	−0.05	96.7	0.90
Environment quality	66.7	0.232	66.7	0.30	86.7	0.62

Intra-rater reliability results were high for all street characteristics, with an average of 81% (k = 0.4) agreement between the first and second audit. The ‘fair’ kappa co-efficient can largely be explained by results from the user conflict category where a result of k = −0.05 was returned despite 90% agreement between measures.

Inter-rater reliability results were high (average 71.7%) for the majority of street characteristics, however, in three categories (pavement quality, lighting, and road permeability) reliability results were low for both percentage agreement (<60%) and the kappa co-efficient (<0.4). Additional detailed analysis revealed that inter-rater reliability varied substantially between land-use types with the lowest average agreement between auditors (60%) recorded in industrial estates and the highest average agreement (74%) attained in residential areas.

Percent agreement between in-person and desk-based audits (criterion reliability) was high across all street characteristic categories with results ranging from 75 to 97% agreement (average 84%) and the kappa co-efficient ranging from k = 0.5 to 0.9 (moderate to almost perfect).

## Discussion

The FASTVIEW tool was found to provide a reliable measure of street characteristics considered likely to be associated with individual walking behaviours. It was found to provide a reliable measure of street characteristics reporting high percentage agreement and moderate kappa coefficients for levels of inter and intra-rater reliability over the majority of street characteristic categories. Furthermore the use of Google Street View to complete street audits provided high levels of agreement between the desk-based and on-site audits.

This new measure is therefore considered to provide a practical and reliable street audit tool. Nevertheless, for three of the nine street characteristic categories, agreement levels were found to be low between auditors. Previous environment audit tools have often noted low levels of inter-rater reliability where variables require a judgement on quality or aesthetics [10,17]. Similarly the lowest inter-rater agreement here was reported for the pavement quality category. Furthermore, when assessed in greater detail, inter-rater reliability was found to vary substantially between land-use types, with the lowest agreement found within industrial estates. Raters had difficulty agreeing on the quality of frontages in industrial areas and whether industrial buildings would provide overnight lighting. Further refinement of the training procedure and guidance manual, in particular focusing on a range of different land-use areas, may be beneficial to ensure high agreement between auditors across all categories.

Findings from this study indicate that desk-based audits using Google Street View provide an effective and reliable alternative to in-person street audits within the UK, with similarly high levels of reliability as those recorded in urban areas of the US [13,14]. This conclusion is important as desk-based auditing can provide many substantial benefits. Most notably the time and costs of objective street auditing are substantially diminished. Indeed trained auditors can now complete large-scale street characteristic research from a single location. Objective analysis is therefore achievable for the majority of research projects, vastly improving the potential for analysis across diverse areas within countries and internationally. Additional benefits of this method include supportive technology such as the distance measurement tool available in Google Street View enabling accurate assessment of variables such as pavement width and street light spacing, which can be difficult to capture in-person, and the removal of any potential lone auditor safety concerns.

### Strengths and limitations

During the auditing process a number of limitations in the use of Google Street View were identified. First, the view from the Google Street View cameras differs to that of a pedestrian auditor, pictures are provided from the centre of the road rather than the pavement, thus, blockages such as parked cars or road works can obstruct views of specific variables on some streets, in particular, pavement quality and signage. Furthermore, image clarity varied dependent upon the weather conditions and lighting when images were taken, and the vehicles used to collect images may not be able to access pedestrianized streets, a particular problem for many city centre audits across the UK. Second, the temporality of Street View can be problematic. For example, results in some categories (e.g. the number of parked cars) may vary throughout the day, but the time when images were taken is not provided by the system. Similarly, although street auditing may be undertaken in conjunction with physical activity assessment, a substantial time lapse may have occurred since pictures were obtained for Street View images, thus the possibility that changes in street conditions may have occurred between measures cannot be ruled-out. Nevertheless the ease, speed, low associated costs and high criterion reliability of desk-based auditing are argued to considerably out-weigh these limitations.

This study provides comprehensive reliability testing of a new audit tool designed specifically for desk-based auditing and the suitability of the measure was tested. One large UK town was chosen for auditing purposes and, following previous recommendations [15], 25% of all possible street links were included in the measure for both inter-rater and intra-rater reliability tests. Previous research assessing the reliability of Google Street View to measure street characteristics has largely focused on urban environments in the US [13,14], this study therefore adds to current research providing results specific to the UK and across a range of land-use types. However, a limitation of the study is that, due to resource restrictions, just 30 street links were audited in-person providing a relatively small sample for criterion reliability analysis. In addition this study did not assess the reliability of the FASTVIEW tool within rural areas of the country.

This research was conducted as part of the wider FAST study measuring environmental associations with adult’s physical activity. Further analysis is necessary to assess associations between street characteristics, measured using the FASTVIEW audit tool, and self-reported walking behaviours and objectively measured physical activity. Further, the value of street audit measures for discriminating walking behaviours over macro level neighbourhood measures, derived from GIS, requires additional analysis.

## Conclusion

The FASTVIEW audit tool provides a reliable, easy to use, and appropriate tool for the objective measurement of street characteristics over a range of differing land-use types and is, therefore, recommended for future large-scale street auditing.

## Abbreviations

FAST: Forty Area STudy; GIS: Geographical Information System; PERS: Pedestrian Environment Review System.

## Competing interests

The authors declare they have no competing interests.

## Authors’ contributions

PG drafted the initial manuscript, PG, MH & CF completed data collection and statistical analysis, EC and AJ completed the GIS mapping. All authors contributed to the design of the project and the writing of the manuscript. All authors read and approved the final manuscript.

## References

[B1] Hamer M Chida Y Walking and primary prevention: a meta-analysis of prospective cohort studies Br J Sports Med 2008 42 4 238 243 10.1136/bjsm.2007.039974 18048441

[B2] Department of Health Physical Activity Health Improvement and Protection Start active, stay active: A report on physical activity from the four home countries' Chief Medical Officers 2011

[B3] Department for Transport National Travel Survey 2010 2011

[B4] Sallis JF Cervero RB Ascher W Henderson KA Kraft MK Kerr J An ecological approach to creating active living communities Annu Rev Public Health 2006 27 297 322 10.1146/annurev.publhealth.27.021405.102100 16533119

[B5] Saelens BE Handy SL Built environment correlates of walking: a review Med Sci Sports Exerc 2008 40 7S S550 S566 18562973 10.1249/MSS.0b013e31817c67a4 PMC2921187

[B6] DeBourdeaudhuij I Teixeira PJ Cardon G Deforche B Environmental and psychosocial correlates of physical activity in Portuguese and Belgian adults Public Health Nutr 2005 8 7 886 895 16277805 10.1079/phn2005735

[B7] Sallis JF Bowles HR Bauman A Ainsworth BE Bull FCL Craig CL Sjostrom M Bourdeaudhuij ID Lefevre J Matsudo V Neighborhood environments and physical activity among adults in 11 countries Am J Prev Med 2009 36 6 484 490 10.1016/j.amepre.2009.01.031 19460656

[B8] Lee C Moudon AV Correlates of walking for transportation or recreation purposes J Phys Act Heal 2006 3 suppl 1 S77 S98 10.1123/jpah.3.s1.s77 28834524

[B9] Purciel M Neckerman KM Lovasi GS Quinn JW Weiss C Bader MDM Ewing R Rundle A Creating and validating GIS measures of urban design for health research J Environ Psychol 2009 29 4 457 466 10.1016/j.jenvp.2009.03.004 22956856 PMC3433081

[B10] Pikora TJ Bull FCL Jamrozik K Knuiman M Giles-Corti B Donovan RJ Developing a reliable audit instrument to measure the physical environment for physical activity Am J Prev Med 2002 23 3 187 194 10.1016/S0749-3797(02)00498-1 12350451

[B11] Dunstan F Weaver N Araya R Bell T Lannon S Lewis G Patterson J Thomas H Jones P Palmer S An observation tool to assist with the assessment of urban residential environments J Environ Psychol 2005 25 293 305 10.1016/j.jenvp.2005.07.004

[B12] Badland HM Opit S Witten K Kearns RA Mavoa S Can virtual streetscape audits reliably replace physical streetscape audits J Urban Health 2010 87 6 1007 1016 10.1007/s11524-010-9505-x 21104331 PMC3005090

[B13] Clarke P Ailshire J Melendez R Bader MDM Morenoff J Using google earth to conduct a neighborhood audit: reliability of a virtual audit instrument Health and Place 2010 16 6 1224 1229 10.1016/j.healthplace.2010.08.007 20797897 PMC2952684

[B14] Rundle A Bader MDM Richards CA Neckerman KM Teitler JO Using google street view to audit neighborhood environments Am J Prev Med 2011 40 1 94 100 10.1016/j.amepre.2010.09.034 21146773 PMC3031144

[B15] McMillan TE Cubbin C Parmenter B Medina AV Lee RE Neighbourhood sampling: how many streets must an auditor walk? Int J Behav Nutr Phys Act 2010 7 20 10.1186/1479-5868-7-20 20226052 PMC3224902

[B16] Sim J Wright CC The kappa statistic in reliability studies: use, interpretation and sample size requirements J Am Phys Ther Assoc 2005 85 257 268 15733050

[B17] Jones N Jones A VanSluijs EMF Panter J Harrison F Griffin SJ School environments and physical activity: the development and testing of an audit tool Health and Place 2010 16 5 776 783 10.1016/j.healthplace.2010.04.002 20435506 PMC3820999

